# Digital Health and Health Systems of the Future

**DOI:** 10.9745/GHSP-D-18-00342

**Published:** 2018-10-10

**Authors:** Alain Labrique, Lavanya Vasudevan, Garrett Mehl, Ellen Rosskam, Adnan A. Hyder

**Affiliations:** a Department of International Health, Johns Hopkins Bloomberg School of Public Health, Baltimore, MD, USA.; b Department of Community and Family Medicine, Duke University School of Medicine, Durham, NC, USA.; c Center for Health Policy and Inequalities Research, Duke Global Health Institute, Durham, NC, USA.; d Department of Reproductive Health and Research, World Health Organization, Geneva, Switzerland.; e Office of the Assistant Director-General, Health Metrics and Measurement, World Health Organization, Geneva, Switzerland.

## Abstract

Digital strategies have been formally recognized as a critical health systems strengthening strategy to help meet the Sustainable Development Goals and universal health coverage targets. This landscaping collection reviews multiple possible approaches across health system pillars, from digital referrals to decision support systems, identifying key knowledge gaps across these domains and recognizing the growth needed in the field to realize its full potential.

## FROM MHEALTH TO DIGITAL HEALTH

Over a decade ago, the emergence of mobile phone networks across the globe presented a novel opportunity for rapid improvement in global health. Although health system challenges were not important drivers of this global mobile network proliferation, the public health and clinical community rapidly recognized the potential of mobile phones to tackle many immense challenges experienced by health systems, including early diagnosis, access to care, and equitable provision of services.^1^ This technology revolution soon became recognized as a way to connect health workers to the people they serve; capture health information, even in hard-to-reach areas; and compress the time between a crisis and an appropriate response.^2^ As is discussed in the opening article of this special issue, the past 10 to 15 years have been characterized by different periods in the evolution of the emergent field of “mHealth”—the common term used to describe mobile phone technologies used in public health or clinical medicine—or digital health, which is our preferred term.

Initially a wide field of discordant experimentation, in the past 5 years digital health has seen an unprecedented convergence on a shared vocabulary,^3^ common tools, and, importantly, principles to guide the selection, implementation, and evaluation of digital innovations. Inspired but also frustrated by the ‘wild west’ character of mHealth innovation, atypical alliances of donors, innovators, governments, and the private sector have emerged in support of common objectives. These alliances and innovations were principally driven by the need to and importance of investing limited human and financial resources into solutions that have value and are robust, scalable, and able to be evaluated. Two key resources were developed to guide digital development and investment: the Principles for Digital Development^4^ and the Digital Investment Principles.^5^ While innovations have been piloted in low- and middle-income countries, concerns about high-income country dominance of digital innovations, inequitable use of and access to digital health technology, and ethical issues related to digital health data remain and continue to be discussed and worked on nationally and globally. The number of digital health programs scaled to the national and subnational levels is growing quickly,^6^ with many countries integrating mobile tools into routine programs to strengthen reporting or boost community health worker performance.

## DIGITAL HEALTH RESOLUTIONS AND GUIDANCE

As we noted previously,^7^ in the early days of mHealth, there was limited enthusiasm and financial support to measure the impact of these interventions through rigorous assessment. The digital exceptionalism that may have colored this early period has since been greatly reduced while, at the same time, a healthy evidence base covering many facets of digital health has grown. To support the development and dissemination of high-quality digital health research, in 2016 the World Health Organization (WHO) mHealth Technical Evidence Review Group published new guidelines^8^ and a toolkit^9^ to help improve and standardize research and reporting of mHealth in the literature.

In May 2018, at the 71st World Health Assembly, WHO member states unanimously endorsed a resolution on digital health^10^ that states governments must recognize the importance of digital systems for facilitating health systems strengthening and achieving universal health coverage. The resolution underscores the need to “ensure that digital health solutions complement and enhance existing health service delivery models,” strengthen already integrated patient-centered health services, contribute to improving population health and gender and health equity, and address the lack of research and evidence on the impact of digital health on public and clinical health.^10^ The resolution identifies coordinated, systematic, and evidence-based approaches that WHO, with member states and the broader ecosystem of partners, will need to prioritize to ensure that the full potential of digital health can be realized.

In May 2018, WHO member states unanimously endorsed a resolution for governments to recognize the importance of digital systems for facilitating health systems strengthening and achieving universal health coverage.

The landmark June 2018 WHO digital health guidelines development meeting and soon-to-be-published guidelines on digital interventions for health systems strengthening^11^ mark a turning point in the digital health ecosystem by addressing another important convergence in the discourse in health systems strengthening and digital health innovation: the consolidation of the evidence-base in the form of global recommendations.

## EXAMINING THE LITERATURE

In this special issue of *Global Health: Science and Practice*, we present 6 articles that explore the ever-growing overlap between health systems and digital health, with each paper led by experts in these 2 domains. Supported by the Aetna Foundation, teams of researchers and practitioners in these often-intersecting domains were asked to use their respective lenses and knowledge of the literature to explore and present an overview of state-of-the-art evidence that illustrates how digital health is being leveraged to address health system constraints across each of the WHO health system building blocks.^12^ Beyond describing key successes, the authors were also asked to identify important roadblocks, such as nascent policy and stewardship architecture, to help guide digital health investments in most low- and middle-income countries. In short, the authors were asked to reflect on key research, policies, and funding priorities within that particular building block.

Labrique et al.^13^ begin the series by summarizing the key milestones marking the journey from the early mHealth pilot studies to the emergence of the digital health ecosystem in 2018. The latter focuses on investments being made in shared resources and the creation and support of a necessary enabling environment for scaling up digital health innovations. The authors assert that greater, concerted investments must be made in the extrinsic ingredients required for digital health scale-up, from establishing national technical standards to bolstering electrical and communications infrastructures at the proverbial last mile, rather than lamenting the fact that so many demonstration projects have failed to thrive. Despite the failures of many pilots, they have, to their credit, generated and continue to develop confidence in novel strategies and solutions. Once tested, however, these innovations need extrinsic enabling systems to allow them to grow and flourish. Frost et al.^14^ build on this theme, exploring the centrality of ministries of health in digital stewardship. Drawing from the literature on successes in policy and leadership, they provide guidance on stewardship responsibilities and the institutional structures and goals needed to meet them.

Meessen^15^ shifts the conversation to the health system's second building block—health care financing—to explore resource generation, resource pooling, and health services purchasing. Using an exciting ‘flash consultation’ approach to poll a global community of implementers, the authors illustrate how digital tools are being leveraged to accomplish these goals using current, real-world examples while also cautioning the reader about the dearth of robust evidence in this space. From innovations in provider selection to smart contract execution and enforcement, Meesen covers the latest innovations and next steps for this domain.

A scoping review of digital technologies that focus on the health workforce, led by Long, Pariyo, and Källander,^16^ assesses current applications in low- and middle-income countries, covering not only the state of the evidence but also the best practices and a research agenda for this space. This team of authors brings many decades of work with community health workers to the table to discuss one of the most researched areas of digital health in health systems strengthening. Through this review, they identify important gaps in best practices and standardized guidelines, which will help lead implementers and governments to develop appropriate digital workforce solutions.

Finally, 2 articles take on the dual aspects of health service delivery—separately tackling digital innovations that target the providers of care (supply-side focus) and the receipt of care (demand-side, client focus). Together with digital health colleagues at WHO, Gibson et al.^17^ review the state of digital demand-generation interventions, highlighting emerging trends in this space. They identify subtleties in the literature that suggest that the contextual tailoring of behavioral interventions by addressing message ‘dose,’ prevalence, and timing may be crucial to program success. Orton et al.^18^ update Agarwal et al.'s^19^ foundational review of mHealth and health worker interventions to include key service delivery research. They identify substantial continuing gaps in current research related to the effectiveness of interventions on health outcomes, improvement in health system efficiencies for service delivery, and the human capacity required to implement and support digital health strategies at scale.

## CONCLUSION

As described earlier, digital strategies have been formally recognized as a critical strategy to help meet the Sustainable Development Goals and universal health coverage targets. As such, this landscaping collection reviews multiple possible approaches, from digital referrals to decision support systems, identifying key knowledge gaps across these domains and recognizing the growth needed in the field to realize its full potential. Strengthening the digital strategy evidence base is crucial, as it provides the necessary support to convince investors and risk-averse governments to invest in this solution space. Across all 6 articles in this supplement, key areas for research, investment, and evidence generation are highlighted, providing a road map for resource allocation in digital health moving forward. An important objective of this series was to identify the ‘big unanswered questions’ that may further the interdisciplinary meeting of health systems strengthening and digital health. The entropy and chaos of the early days of digital health is rapidly decreasing, guided by strong leadership, clear visions, and key investments in ‘global goods.’^20^ With measured optimism, we see the coming decade continuing the trend of thoughtful experimentation, planning for scale and sustainability, and cooperation.

Strengthening the digital strategy evidence base is crucial, as it provides the necessary support to convince investors and risk-averse governments to invest in this solution space.

## Notes

**Funding:** This work was made possible by a grant from the Aetna Foundation, with support from the World Health Organization and The Johns Hopkins University Global mHealth Initiative.
